# Cancer Treatment in CKD: Time to Move Beyond Renalism

**DOI:** 10.34067/KID.0000000000000258

**Published:** 2023-09-28

**Authors:** Joseph Rossi Berger, Miguel Angel Vazquez

**Affiliations:** UT Southwestern: The University of Texas Southwestern Medical Center, Dallas, Texas

**Keywords:** cancer, CKD, cisplatin nephrotoxicity, clinical nephrology, drug nephrotoxicity

The link between kidney disease and cancer is complex. AKI and CKD are more common in patients with cancer, and kidney disease often affects treatment selection for patients with cancer.^1^ Urothelial cancer is one of the most common cancers in the world and frequently diagnosed in patients with CKD.^2^ Urothelial cancer can contribute to significant morbidity and mortality in patients with metastatic disease. Concerns about nephrotoxicity from chemotherapy play a key role in treatment selection for urothelial cancer.

In this issue of *Kidney360*, Cote *et al.*, describe a single-center experience with the treatment of metastatic urothelial carcinoma (mUC). Cisplatin-based chemotherapy is considered the first-line treatment of mUC. Patients with kidney disease are frequently deemed ineligible for cisplatin because of kidney disease. In this retrospective study, the authors compared outcomes for patients with mUC treated with standard-dose gemcitabine-cisplatin, split-dose cisplatin, and a noncisplatin-based regimen.^3^ There was no difference in the occurrence of AKI among the three groups.^3^ When AKI occurred, it was most commonly mild (Kidney Disease Improving Global Outcomes stage 1). Patients who did not receive full-dose cisplatin had a lower eGFR than those who received standard therapy, and lower kidney function was a frequent reason for not receiving standard therapy. Unfortunately, patients who did not receive the standard regimen had significantly worse survival compared with those who did receive standard treatment.^3^ This study has various limitations as noted by the authors. It was a retrospective study, and there is a risk of selection bias, and the unaccounted confounders, such as patient comorbidities or poorer functional status, associated with CKD may have affected treatment selection and results. The overall sample size was small. Few patients had laboratory values available to assess long-term kidney function. There was no information available on concurrent exposure to other medications.

Even with the above limitations, the study does present the very important observation that many patients did not receive the best therapy for mUC because they had kidney disease. Limiting patient access to best treatment options solely because of the presence of kidney disease is a form of renalism. This term was first used almost two decades ago by Chertow *et al.*, to describe the systematic withholding of indicated, potentially beneficial studies and interventions from patients on the basis of coexisting kidney disease.^4^ This was initially described in the context of cardiovascular disease, specifically decreased rates of coronary angiography after myocardial infarction for elderly (age 65–89 years) patients with kidney disease compared with those with normal kidney function.^4^ Similarly, patients with kidney disease are less likely to receive cardioprotective medications than those with normal kidney function.^5^

Treatment of cancer for patients with kidney disease represents another example of renalism. Although CKD is highly prevalent among those receiving treatment for cancer, these patients are grossly underrepresented in clinical trials of cancer therapy with 85%–95% of major anticancer trials excluding a majority of patients with CKD and with very strict kidney function eligibility criteria.^6,7^ There are various reasons for reluctance to include kidney patients in cancer therapy trials.^8^ Many anticancer therapies are cleared by the kidney or may have nephrotoxicity.^7^ Excluding patients with kidney disease may reflect a desire to enhance the likelihood for a drug to move through the development, testing and approval process (by minimizing chances of adverse events), concern over contrast-based radiographic studies for disease monitoring, and safety concerns.^8^ Patients are frequently excluded with only very mild kidney disease, many times on the basis of a serum creatinine alone as measurement of kidney function, and often distinction is not made for drugs that do not rely on the kidney for metabolism or clearance.^7,8^ The impact of excluding patients with CKD from cancer therapy trials is further magnified in the aging population with higher risk for coexistent cancer and kidney disease.^8^ For some of these older patients, renalism and ageism, excluding patients from diagnostic and therapeutic interventions on the basis of chronologic age, combine to further limit access to care.

As a result of the limited access of kidney patients to cancer therapy trials, there is limited information to guide clinicians on how to safely use these treatments for patients with kidney disease. Consequently, many patients with kidney disease are denied access to cancer treatments which may have led to improved outcomes. The findings reported by Cote *et al.*, provide such a clear example of renalism and its adverse effects on care for patients with mUC.

What can be done to lessen the impact of renalism? The arena of drug development and clinical trials should be targets for improvement. Awareness of renalism exists, and efforts have been made for improved representation of multiple stakeholders in trial designs for patients with cancer and kidney disease. The input of patients, oncologists, nephrologists, and pharmacists working closely with professional societies, pharmaceutical companies, and regulatory agencies is key to design clinical trials that can inform the field.^8^ The American Society of Clinical Oncology has issued guidance to improve participation in clinical trials for patients with comorbid conditions, including kidney disease.^9^ The US Food and Drug Administration has also provided guidance meant to enhance inclusion of patients with CKD in randomized controlled drug trials.^10^

Important steps include reassessing kidney eligibility criteria for cancer trials with more precise determinations of kidney function and pharmacokinetic studies of anticancer drugs in kidney models.^8^ Large-scale observational studies across a larger spectrum of primary cancers can help to shed additional light on the problem and add to the planning of clinical trials and advance collection of valuable data. Other steps include leveraging data from electronic health records to reach larger pools of potential participants and trial designs.^7,8,10^ Continued and intensified advocacy for consideration of patients with CKD in cancer therapy trials is essential and has been successful in other fields. This is a proven and direct way to bring additional resources and support studies to allow advances and generate the evidence to guide treatment decisions.

An interesting observation from the study by Cote *et al.*, is that a small but significant number of patients in the standard treatment arm received full-dose cisplatin despite an eGFR <60 ml/min per m^2^.^3^ This represents some deviation by some of the oncologists from conventional practice. Did these providers make the decision unilaterally? Did they seek input from nephrologists? This highlights the opportunity for nephrologists and other clinicians and investigators to make a day-to-day impact through simple communication and collaboration.

Health care professionals also have an opportunity to make an impact in medical education. It is likely that renalism has made its way into the larger curriculum of medical students, residents, fellows, scientists, and pharmacists. It is not difficult to envision learners accepting the premise that some indicated treatments are not available to kidney patients. In this context, there is an opportunity to role model patient advocacy.

In conclusion, renalism is a real barrier to patient care. As reported by Cote *et al.*, ineligibility to access the best therapy for mUC on the basis of concerns about reduced kidney function was associated with higher risk of progressive disease and reduced patient survival.^3^ There is clearly a need to revisit cisplatin eligibility criteria and develop new strategies such as immunotherapy, targeted treatments, and other novel therapies for patients with mUC and kidney disease.^11^ Moreover, there is an opportunity to revise the broad restrictions on access to cancer therapies in general for patients with CKD. It is now time to collect the necessary data and develop an evidence-based approach to guide development of best care practices for patients with cancer and kidney disease.
